# Alimentary tract obstruction attributed to use of barbed suture for double tract reconstruction after robot-assisted proximal gastrectomy: a case report

**DOI:** 10.1186/s12893-021-01407-9

**Published:** 2021-11-29

**Authors:** Daisuke Fujimoto, Keizo Taniguchi, Fumihiko Miura, Hirotoshi Kobayashi

**Affiliations:** Department of Surgery, Teikyo University Hospital Mizunokuchi, 5-1-1 Futako, Takatsu-ku, Kawasaki City, Kanagawa 213-8507 Japan

**Keywords:** Barbed suture, Overlap method, Robot-assisted surgery, Tactile sensation

## Abstract

**Background:**

Anastomotic stenosis following esophagojejunostomy reconstruction by the overlap method with absorbable barbed sutures occurs only rarely in patients who have undergone laparoscopic surgery. We report anastomotic stenosis by the overlap method that we attributed to the lack of tactile sensation during robot-assisted surgery.

**Case presentation:**

An 83-year-old man underwent robot-assisted laparoscopic proximal gastrectomy and lymph node dissection at our hospital for treatment of gastric cancer. Double tract reconstruction followed with side-to-side esophagojejunostomy (overlap method) performed with an endoscopic linear stapler. On completion of the anastomosis, the enterotomy was closed under robotic assistance with absorbable barbed suture. Once solid foods were introduced, the patient had difficulty swallowing and felt as though his digestive tract was stopped up. When upper gastrointestinal endoscopy was performed, we found the anastomotic lumen to be coated with food residue. After rinsing off the residue with water, we could see barbed suture protruding into the anastomotic lumen that had become entangled upon itself, which explained how the food residue had accumulated. We cut the entangled suture under endoscopic visualization using a loop cutter.

**Conclusion:**

This case highlights a stricture caused by insufficiently tensioning barbed suture, which subsequently protruded into the anastomotic lumen and became entangled upon itself. We believe this occurrence was associated with the lack of tactile sensation in robot-assisted surgery.

## Background

Esophagojejunostomy reconstruction by the overlap method is commonly performed in laparoscopic proximal or total gastrectomy, and enterotomy closure is often performed using absorbable barbed suture. Reported cases of stenosis are extremely rare [1–5]. Absorbable barbed suture with microwings and a terminal ring is intended to pass through the tissue in one direction to secure the tissues without knot-tying, thus ensuring that suture loosening does not occur. The safety of barbed suture was shown in a series of patients who underwent enteroanastomosis; the suture retains about 50% tensile strength after 21 days, and complete absorption occurs within 180 days [6]. Only a single case of predischarge anastomotic stenosis has been reported in association with esophagojejunostomy construction by the overlap method and use of absorbable barbed suture, and the stenosis improved without balloon dilation [3]. Here, we highlight our patient with anastomotic stenosis in whom the stricture was caused by not sufficiently pulling on the barbed suture, which subsequently protruded into the anastomotic lumen and entangled upon itself, due to the lack of tactile sensation in robot-assisted surgery.

## Case presentation

**Fig. 1:**
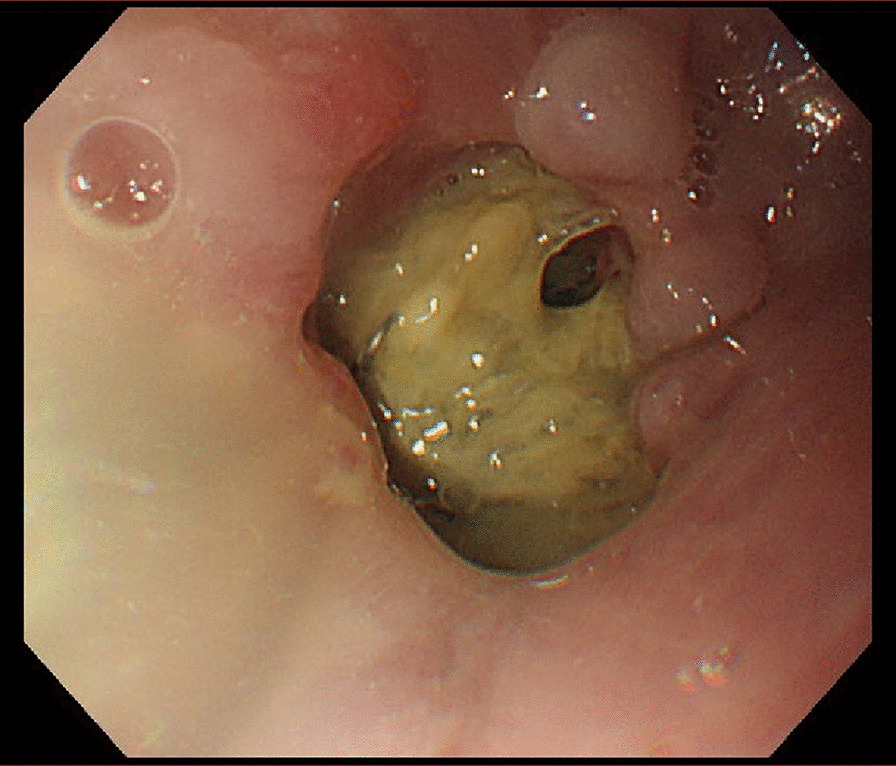
Upper endoscopic examination performed 2 weeks after the surgery revealed obstruction at the site of esophagojejunal anastomosis due to food residue

**Fig. 2:**
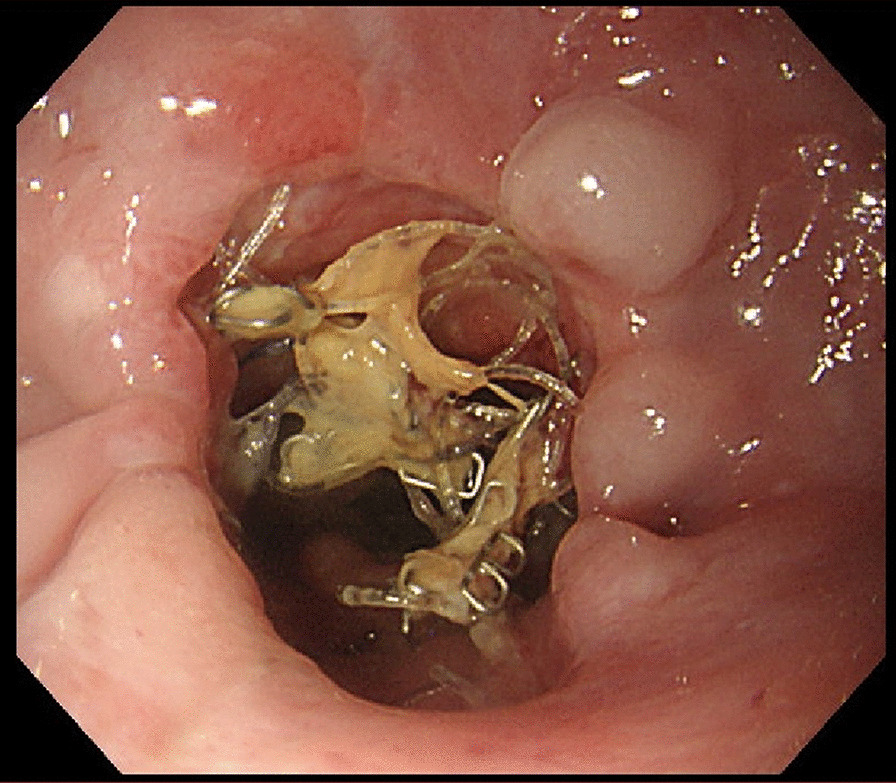
When the residue was rinsed away, barbed suture protruding into the anastomotic lumen came into view

An 83-year-old man underwent radical gastrectomy at our hospital for treatment of gastric cancer. Because the tumor was located just below the gastric cardia and clinical staged as cT2N0M0 and stage I according to the American Joint Committee on Cancer 8th edition, robot-assisted laparoscopic proximal gastrectomy and lymph node dissection were performed. Double-tract reconstruction followed, with side-to-side esophagojejunostomy (overlap method) performed with an endoscopic linear stapler [1]. On completion of the anastomosis, the enterotomy was closed under robot assistance in a full-thickness layer with absorbable barbed suture of 15 cm in length. The patient’s early postoperative course was good, and he had no trouble ingesting fluids or a liquid diet. However, once solid foods were introduced, he had difficulty swallowing and felt as though his digestive tract was stopped up. This reaction persisted, and the clear liquid diet was resumed. Two weeks after the gastrectomy, upper gastrointestinal endoscopy was performed to confirm alimentary tract continuity. We found the anastomotic lumen to be coated with food residue (Fig. 1). After we rinsed off the residue with water, we could see barbed suture protruding into the anastomotic lumen that was entangled upon itself, thus explaining how the food residue had accumulated (Fig. 2).

**Fig. 3:**
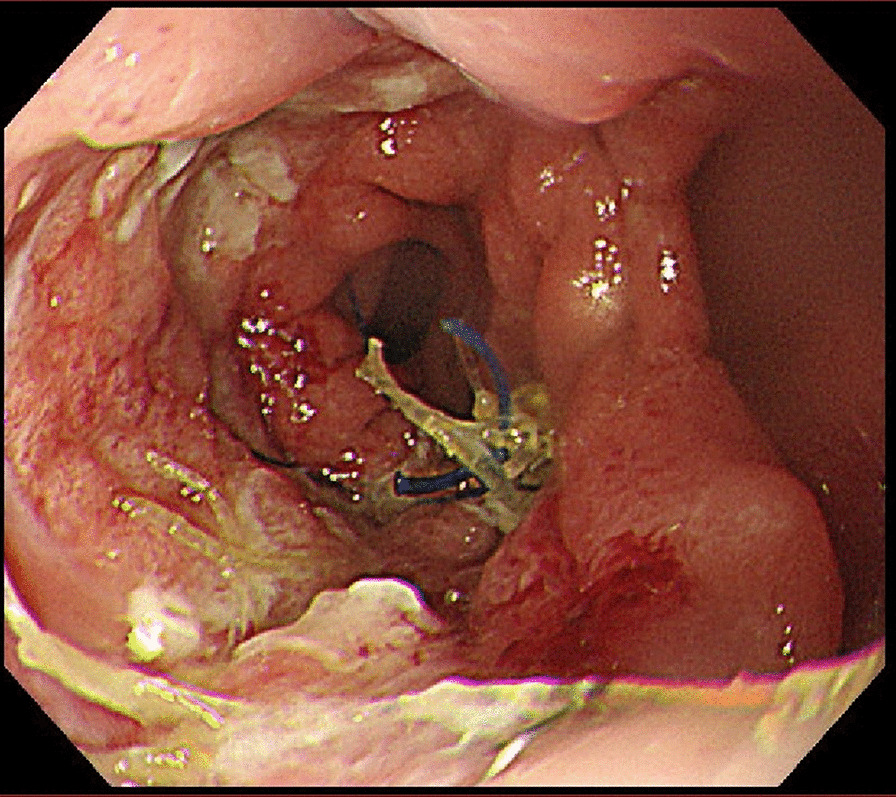
The protruding barbed suture was cut to secure the suture in place

Under endoscopic visualization, we cut the entangled suture using a loop cutter (Fig. 3). Over the next couple of days, the patient’s intake of solids increased normally, and he was discharged 3 days after the endoscopic treatment. He has had no particular symptoms such as dysphagia.

## Discussion and conclusion

Anastomotic stenosis following overlap reconstruction occurs only rarely in patients who have undergone laparoscopic surgery [1–5]. The stricture in our robot-assisted surgical case was caused by protrusion of the barbed suture into the anastomotic lumen and subsequent entanglement, an occurrence that might have been due in part to the lack of tactile sensation experienced during the robot-assisted surgery.

Robot-assisted surgery has not only restored the surgeon’s natural 3-dimensional vision but has also improved the surgeon’s skills by allowing fluid movements while eliminating the inevitable tremors and shaking of the surgeon’s hand and permitting increased reproducibility of movements [7, 8]. Additionally, the articulation of the robotic arms during instrument use imitates that of the wrist, thus making triangulation in confined spaces more realistic and intracorporeal suturing almost a natural task [9]. However, the main limitation of robotic technology even today is the loss of tactile sensation and feedback [10], which can potentially result in collateral injury and unexpected complications [10]. There are few reports of cases of predischarge anastomotic stenosis by the overlap method (1–5). Tactile sensation is present in laparoscopic surgery, although it is reduced compared to that in open surgery. However, tactile sensation is absent in robotic surgery, and thus, the surgeon adjusts the power applied to pulling on sutures using visual information alone. We think that this case clearly suggests that visual information alone is insufficient. Although the surgeon determined that he had pulled on the suture with sufficient and appropriate power, in fact, the suture was not sufficiently tensioned, and it became entangled in the lumen of the anastomosis.

We believe that this case should cause surgeons to realize the importance of overcoming the lack of tactile sensation, which is the present situation in robot-assisted surgery.

## Data Availability

This patient data and clinical images adopted are contained in the medical files of Teikyo University Hospital, Mizonokuchi, Japan. All data generated or analyzed during this study are included in this published article. The datasets used during this study are available from the corresponding author on reasonable request.
